# The Problem of Radiant Heat v. Warmed Air

**Published:** 1906-04-21

**Authors:** 


### THE PROBLEM OF RADIANT HEAT v. WARMED AIR.

Strictly speaking, radiant heat should cover all heat emanating from any source, provided that it is given off by radiation. Thus the pipes that are sometimes employed to warm rooms, and the radiators that are now so common, both of which are either dull black or painted in accordance with the decorations of the room, give out heat by radiation. But when one refers to radiant heat, one has in one's mind the heat given out by a glowing fire of coals or wood logs, and it is in this sense that the term will be used in this article. There is a distinct difference between the emanations from a glowing fire and those from a dull black mass of metal, quite apart from the fact that radiators are used in the latest practice more to warm air than to radiate heat. The glowing fire gives out light as well as heat, and if the latest researches on the subject are worth anything, if the common experience of mankind is worth anything, light plays as important a part in health giving as heat. A coal fire is not the sun, but it is a nearer approach to the sun than a current of warmed air. Everyone who has a choice prefers to warm himself by a bright fire than by means of a warm air current; and it is a striking commentary on modern developments that in the Western States of America the writer understands they are arranging in all recently built houses for coal fires to be used until the very cold weather sets in. The method of warming buildings by warming the air that is used for ventilating them has been gradually developed, first in America and Canada, because the heating problem there was involved in a twofold difficulty. The cold is very intense for a good many months of the year, and domestic labour is very scarce. It was a natural consequence of the apparent inability of the open fire to efficiently warm living rooms of any size. As the advocates of warmed air point out, the greater part of the heat furnished by the combustion of the coal is carried up the chimney with the products of combustion. The open fire is also subject to the disadvantage that its heating effect decreases very rapidly as one moves away from it. At six feet the heating effect is only one-fourth that at three feet, while at nine feet it would be only one-ninth. Again, physicists tell us that air is transparent to radiant heat. This is not quite correct. They apparently mean that the air through which the radiations pass is not heated by them. The air is heated, though not to such an extent as when it is passed over a heated surface. The actual behaviour of air in the presence of heat is not thoroughly understood. It resists the passage of heat through it very strongly so long as it is not in motion, but immediately any portion moves it becomes a very good heat dissipator, the convection currents that are set up effectively transporting large quantities of heat. The heat from open fireplaces acts by warming the surfaces of the objects and the walls of the room—by setting up convection currents in the air in contact with those objects, and by reflection from them, the latter operation giving rise to further convection currents; so that, provided the area of a room to be warmed is not greater than a certain figure, and provided that there are heat absorbing objects in the room, such as beds, chairs, and other furniture, it is perfectly possible to warm a room by a coal fire quite as well as by any system of air warming; and the problem resolves itself, therefore, where the rooms are large, into the provision of a sufficient number of fires to cover the whole of the ward, or whatever the room may be, and this again has been rendered quite practicable by the improvements in the modern fireplace. It is no longer necessary to place the fire at one end of the room. There may be as many as are necessary in different parts of the room, as in the new wards at Charing Cross Hospital. The modern double stoves, with two fires looking in opposite directions, should furnish very efficient heating. In addition, the modern fireplace does not allow all the heat to pass up the chimney. It is arranged, even in grates fitted in comparatively small houses, to reflect a much larger portion of the heat out into the room than the old fireplace did, and further, many of them are arranged to provide a current of fresh warm air, coming from outside the building, and warmed by passing over the back of the flue of the fireplace. If the heating problem be taken to consist of two parts, heating the human body as a whole and heating the air that is breathed, heating the body must be effected in a better manner by the direct rays from the glow of the fire—it is common experience that this is so—than at second hand, through the medium of air, heated by passing over a steam pipe, and any heating of the air that is wanted in addition should be provided by the air warming arrangements of the stoves described. Air heating on a large scale, as in the Plenum system, has also the disadvantage that is common to all machine products, it is too uniform, while men and women are not, especially when ill. It is possible to conceive that an amputating machine would expedite matters very much on a battlefield, and the man who had an average limb, which was injured in the average way, that for which the machine was constructed, might even come off better than if operated on by the average army doctor; but few of us are average, and fewer are injured in the average manner. Similarly it appears to the writer, while giving the Plenum system full credit for what it has achieved, that its success is obtained at the cost the individuality that is of such enormous importance in medical and surgical work. Different wards may require different temperatures, and different degrees of humidity of the atmosphere, at different times of the day, and not only so, but different portions of a particular ward may do so. This, it appears to him, is possible with modern fireplaces—but only with great difficulty, if at all, by any system of warming the air of several wards together.

